# Precision Tracing of Household Dengue Spread Using Inter- and Intra-Host Viral Variation Data, Kamphaeng Phet, Thailand

**DOI:** 10.3201/eid2706.204323

**Published:** 2021-06

**Authors:** Irina Maljkovic Berry, Melanie C. Melendrez, Simon Pollett, Katherine Figueroa, Darunee Buddhari, Chonticha Klungthong, Ananda Nisalak, Michael Panciera, Butsaya Thaisomboonsuk, Tao Li, Tyghe G. Vallard, Louis Macareo, In-Kyu Yoon, Stephen J. Thomas, Timothy Endy, Richard G. Jarman

**Affiliations:** Walter Reed Army Institute of Research Viral Diseases Branch, Silver Spring, Maryland, USA (I. Maljkovic Berry, M.C. Melendrez, S. Pollett, K. Figueroa, M. Panciera, T. Li, T.G. Vallard, R.G. Jarman);; Armed Forces Research Institute of Medical Sciences, Bangkok, Thailand (D. Buddhari, C. Klungthong, A. Nisalak, B. Thaisomboonsuk, L. Macareo);; International Vaccine Institute, Seoul, South Korea (I.-K. Yoon);; Upstate Medical University of New York Department of Medicine, Syracuse, New York, USA (S.J. Thomas, T. Endy)

**Keywords:** arboviruses, viruses, dengue, transmission chains, intra-host variants, inter-host variants, mosquitoborne diseases, vector-borne infections, Kamphaeng Phet, Thailand

## Abstract

Dengue control approaches are best informed by granular spatial epidemiology of these viruses, yet reconstruction of inter- and intra-household transmissions is limited when analyzing case count, serologic, or genomic consensus sequence data. To determine viral spread on a finer spatial scale, we extended phylogenomic discrete trait analyses to reconstructions of house-to-house transmissions within a prospective cluster study in Kamphaeng Phet, Thailand. For additional resolution and transmission confirmation, we mapped dengue intra-host single nucleotide variants on the taxa of these time-scaled phylogenies. This approach confirmed 19 household transmissions and revealed that dengue disperses an average of 70 m per day between households in these communities. We describe an evolutionary biology framework for the resolution of dengue transmissions that cannot be differentiated based on epidemiologic and consensus genome data alone. This framework can be used as a public health tool to inform control approaches and enable precise tracing of dengue transmissions.

Dengue virus (DENV) causes an estimated 390 million infections each year, 96 million of which manifest as clinical disease (*1*). Dengue is endemic in >100 countries. An estimated 3.9 billion persons are at risk for infection, of which 75% reside in the Asia-Pacific region (*2*). DENV control approaches are best informed by granular spatial epidemiology of these viruses, and investigators have long tried to understand the landscape of DENV dynamics and spread. Robust public health surveillance and rigorous academic research have enabled tracking of clinical infections to understand changing demographics, populations at risk, hyperendemicity, transmission, disease severity, and virus dispersal patterns within and across human populations (*3*–*10*). With the advent of sequencing technologies, single-gene and whole-genome DENV analyses have complemented case-based and serologic surveillance and enabled further insights into DENV epidemic dynamics and spread, disease severity, introductions and emergence of novel variants, tracking of DENV transmissions, and monitoring of viral diversity and evolution (*11*–*17*). Investigations of DENV infection dynamics, from the scales of countries and districts down to the city, village, school, and house level, have been performed to describe the patterns and predictors of DENV spread (*7*,*9*,*11*–*13*,*18*–*21*). These studies have enabled many novel insights into DENV transmission and contributed to improved prediction, prevention, and control strategies. Such studies have also emphasized a key limitation of DENV genomic epidemiology: reconstructing transmission chains between households and within households is typically not possible, even though resolving DENV spread at such fine scales would offer major relevance to public health response.

Consensus whole-genome sequences, which are typically used in genomic studies of viral epidemics and spread, typically have insufficient variability to distinguish between infecting strains sampled within 2 weeks of each other, especially if closely sampled in space (*22*). This limitation might lead to unresolved and low confidence phylogenetic tree topologies, unable to discern the exact relationships between closely related taxa, and thus unable to provide confident insights into the fine scale viral transmission patterns. In the case of DENV, the use of consensus env-gene or whole-genome data has been able to resolve fine-scale clustering of cases within 200 m but has not been able to determine discrete inter-household or within-household transmissions (*12*).

DENV, like other RNA viruses, exists within a host as a population of distinct viral variants. Together, these viral variants are usually assembled into a single viral consensus genome, but separately, they hold additional intra-host variant sequence information that can provide further resolution. Transmission of within-host minor viral variants, existing at low frequencies and therefore often not reflected in the consensus genome, has been reported for several different viral pathogens (*23*–*27*). An increasing number of studies have used this additional genetic information to confirm viral transmissions, and new tools that use both intra- and inter-host genetic variation have been developed for inference of transmission chains and transmission directions (*23*,*26*,*28*,*29*). We designed a study that explicitly measures the patterns of DENV minor variant transmission in a natural epidemic setting, to resolve more spatially explicit viral transmissions.

We investigated the level of transmission reconstruction granularity that can be achieved for DENV by analyzing 410 DENV whole genomes sampled from human and vector specimens in Kamphaeng Phet, Thailand, and sequenced by using next-generation sequencing techniques. A combination of Bayesian analyses and intra-host single nucleotide variant (iSNV) information, along with temporal and spatial epidemiologic data, enabled fine-scale reconstructions of transmission chains and house-to-house spread, as well as genomic confirmation of within-household transmission clusters. This approach could be used in public health to reconstruct fine-scale DENV transmissions and support dengue control and prevention efforts.

## Methods

### Ethics Statement

The samples used in this study were virus isolates, which originated from samples collected in a study described in Thomas et al. (*30*), per the protocols approved by the institutional review boards (IRBs) of the Thai Ministry of Public Health, Walter Reed Army Institute of Research, and the State University of New York’s Upstate Medical University. The IRBs of the University of California, Davis, University of Rhode Island, and University at Buffalo established relying agreements with the Walter Reed Army Institute of Research IRB. All isolates were deidentified, and this study’s research team had no access to identification codes.

### Prospective Cluster Study and Data Collection

The prospective cluster study method has been described previously (*30*). In brief, the study was conducted in Kamphaeng Phet Province (Kamphaeng Phet), Thailand. A blood sample was obtained from patients admitted to Kamphaeng Phet Hospital with a diagnosis of an acute dengue infection; if positive for DENV by PCR, the virus was isolated with low passage number in C6/36 cell culture or in Toxorhynchites splendens mosquito followed by C6/36 (Appendix Table 1). Patients who were DENV PCR-positive were considered index case-patients for a cluster investigation. The exact house locations of index case-patients was mapped through a geographic information system by using a global positioning system unit. Homes within 100–200 m radius around the index case-patient’s house were screened for additional cases in persons with a history of temperature >38°C within past 7 days, and a blood sample was collected from those persons who agreed to participate in the study. DENV reverse transcription PCR, viral isolation, and IgM/IgG ELISA were performed on all collected specimens.

### Sequencing, Genome Assembly, and Minor Variant Determination

We sequenced all DENV isolates to obtain whole genomes during 2009–2012 by using the Roche 454 FLX system (Roche, https://www.roche.com), the Illumina MiSeq next-generation sequencing system (Illumina, https://www.illumina.com), and, for gap filling, the Applied Biosystems 3130 Sanger sequencing platform (ThermoFisher Scientific, https://www.thermofisher.com). We generated consensus genomes by using the in-house developed ngs_mapper reference mapping pipeline and manually curated to ensure consensus accuracy (*31*). We submitted all consensus genomes to GenBank (accession nos. MN448597–9006). In addition to consensus, we screened all DENV serotype 1 (DENV-1) MiSeq-derived assemblies that had a minimum 1,000× depth of coverage throughout the genome for presence of iSNVs. We excluded DENV serotype 2 (DENV-2) genomes from iSNV analyses because some of the samples did not have enough coverage, mainly because of being sequenced by using Roche 454. iSNVs were called if they were present at a conservative frequency of >1% (minimum Phred of 30) called by Lofreq, a base caller previously shown to confidently call DENV iSNVs at this frequency (*32*,*33*). Additional manual iSNV curation removed variants present because of primer induced error, certain types of sequencing error, and strand bias higher than what was observed in confident iSNVs from each genome. In addition, we manually removed iSNVs that were consistently present at either ends of the reads from the analyses because these iSNVs have previously been shown to be spurious (*33*).

### Phylodynamic Analyses

We combined genomes for DENV serotypes 1–4 with GenBank references and aligned by using MUSCLE 3.8 (*34*). We used jModeltest2 to determine the best fit model of nucleotide substitution (*35*) and constructed maximum-likelihood trees for whole-genome sequence data from all 4 serotypes by using PhyML 3.0 with aLRT node support (*36*). Because discrete phylogeographic analyses to infer the household-scale geographic histories of all sampled viruses were computationally unfeasible, we subselected datasets from DENV maximum-likelihood trees that had most taxa sampled from households in the same subdistrict and had sufficient temporal structure (root-tip regression r>0.7) as determined by Temp-Est (*37*). Using these criteria, we selected 2 DENV-1 sublineages and 3 DENV-2 sublineages to infer the inter-household patterns of DENV spread by using BEAST 1.8.4 (*38*). We excluded DENV serotypes 3 and 4 (DENV-3 and DENV-4) from further analyses because of the weak sublineage temporal structure and limited clade sizes by individual subdistricts of Kamphaeng Phet. For each sublineage analysis, we geotagged time-stamped taxa by household as discrete traits. The final Markov Chain Monte Carlo chains had lengths required for statistical convergence, as indicated by effective sample size values >200 for key evolutionary parameters (Appendix Table 2). We constructed and annotated maximum clade credibility (MCC) trees by using TreeAnnotator. We manually inspected the MCC trees to determine probable and possible inter-household transmissions events. We defined probable inter-household transmissions as origin household being directly ancestral to the destination household and both geographic states supported with a probability >0.8 and plotted all iSNVs onto the resulting MCC trees. We used this information for confirmation or detection of between-household spread and for detection of within-household connections.

### iSNV Distribution Statistical Analyses

We compared the frequency of infected case-patients sharing iSNVs within a transmission cluster with the frequency of samples sharing these iSNVs across the sublineage by using χ^2^ test. We defined a transmission cluster as the smallest cluster of genomes in the MCC tree containing all the genomes sharing the same iSNVs. Thus, if shared iSNVs are distributed across the tree in an unrelated manner, the transmission cluster they define will be large and will not differ significantly from the whole sublineage tree. A significant difference from the sublineage tree would thus indicate iSNV clustering not expected by chance. In addition, we compared pairwise p-distances between the genomes sharing iSNVs with 100 replicates of pairwise p-distances between 3 randomly sampled genomes across the 2 DENV-1 sublineages by using an in-house developed script. Using 3 genome pairwise distance in the randomized dataset was determined on the basis of the average size of the significant transmission clusters, as defined by χ^2^ test.

### Linear Regression Analyses of Transmission-Pair Distance over Sampling Time

We performed crude estimates of the dispersal rate of DENV at an inter-household scale by a univariate linear regression model, which fit the difference in sampling days between confirmed transmission pairs as a single predictor, and distance between confirmed transmission pairs as the outcome variable. We fit this model by using all transmission pairs confirmed by the Bayesian consensus analysis, the iSNV analyses, or both. We used the coefficient of determination (R^2^) to determine model fit and the coefficient point estimate and 95% CI to estimate the average rate of DENV spread between households. We performed all regression analyses by using Stata 15.1 (StataCorp, https://www.stata.com).

## Results

PCR-confirmed DENV cases in Kamphaeng Phet during 2010–2012 were dominated by DENV-2, with co-circulating DENV-1 and DENV-3. DENV-4 was detected to a small degree in 2011 and 2012 (Appendix Figure 1). A total of 410 DENV whole genomes were sequenced from samples collected during 2009–2012 in Kamphaeng Phet, including 100 DENV-1, 233 DENV-2, 64 DENV-3, and 13 DENV-4 genomes. The unique design of the Kamphaeng Phet cluster study provided an opportunity to investigate fine-scale DENV spread between households in Kamphaeng Phet. Analyses of DENV serotype 1–4 phylogenetic trees identified 2 DENV-1 and 3 DENV-2 sublineages with adequate temporal structure and sufficient sample size for Bayesian analyses. DENV consensus genomes from DENV-1 and DENV-2 sublineages geotagged by their respective household of sampling were used as discrete traits for Bayesian ancestral state reconstructions. We confirmed between-household direct connections with high probability in 4 DENV-1 cases and 3 DENV-2 cases, in 7 different subdistricts of Kamphaeng Phet (Table 1; Appendix Figures 2, 3).

**Table 1:** Summary of probable household-to-household spread of dengue virus inferred by consensus genomes, Kamphaeng Phet, Thailand, 2009–2012

Serotype and sublineage	Subdistrict	Origin household† (location probability)	Destination household (location probability)	Approximate distance, m	Sampling time difference, d
DENV-1, sublineage 1	LD	LD02H075 (1.0)	LD01H116 (1.0)	1,400	19
DENV-1, sublineage 1	SK	SK06H346 (0.99)	SK06H370 (1.0)	3,000	29
DENV-1, sublineage 7	TN	TN18H021 (0.88)	TN18H014 (1.0)	80	1
DENV-1, sublineage 7	NC	NC06H057 (0.82)	NC06H407 (1.0)	30	0, 14‡
DENV-2, sublineage 2	NB	NB06H055 (0.80)	NB06H084 (1.0)	800	13
DENV-2, sublineage 2	NP	NP08H080 (0.82)	NP08H044 (1.0)	180	0
DENV-2, sublineage 6	SK	SK06H790 (0.99)	SK06H485 (1.0)	3,000	49

*LD, Lan Dokmai; NB, Na Bo Kham; NC, Nakhon Chum; NP, Nong Pling; SK, Sa Kaeo; TN, Thep Nakhon. †Households are described by their subdistrict, cluster, and house numbers, such that the first 2 letters denote subdistrict, the next 2-digit number denotes cluster, and an H followed by a 3-digit number denotes house number. ‡Two persons from the donor household were dengue virus–positive 2 weeks apart.‡

These analyses also indicated many weakly supported inter-household transmissions (probability of origin or destination household <0.8) (Appendix Table 3). This finding highlighted the limited spatial and temporal resolution that is obtained by consensus genome data only, and prompted an analysis of iSNVs.

We determined iSNVs for each of the DENV-1 genomes and plotted on the sublineage MCC trees of DENV-1. Closely related viruses in the MCC phylogenies often shared the same iSNV spectra, including >2 shared iSNVs (Appendix Figure 2). In total, this analysis revealed 3 transmission clusters that involved shared iSNVs, revealing direct DENV spread between households that were 20–800 m apart and involved a total of 11 persons (Table 2; Figure). The clusters contained persons from both separate and same households. The transmission confirmed by BEAST in transmission cluster 3 consisted of viruses with very little within-host variation (0 and 1 iSNV) (Appendix Figure 2), indicating that close transmission connections were not always characterized by iSNV sharing. In addition, this transmission cluster showed how iSNVs can be lost in bigger clusters over time, such as the loss of 1853-C/T iSNV in 1 person from household TN18H023 (Appendix Figure 2). Thus, the presence of >2 shared iSNVs indicated fine-scale virus transmission connections, whereas absence of >2 shared iSNVs did not imply lack of shared connection.

**Table 2:** Dengue virus serotype 1 transmission clusters revealed by minor variant sharing, Kamphaeng Phet, Thailand, 2009–2012*

Sublineage	Transmission cluster	Household†	No. persons	Sampling dates	Approximate distance, m
1	1	LD02H056	1	2011 Jun 2	800
		LD10H001	1	2011 Jun 16	
7	2	ST05H002	2	2011 Nov 28–29	0
7	3	TN18H023	3	2012 Oct 10	0–80
		TN18H019	1	2012 Oct 10	
		TN18H021‡	2	2012 Oct 10	
		TN18H014‡	1	2012 Oct 9	

*LD, Lan Dokmai; ST, Song Tham; TN, Thep Nakhon. †Households are described by their subdistrict, cluster, and house numbers, such that the first 2 letters denote subdistrict, the next 2-digit number denotes cluster, and an H followed by a 3-digit number denotes house number. ‡House or individual involvement with the transmission cluster also confirmed by BEAST analyses (*38*).

**Figure:**
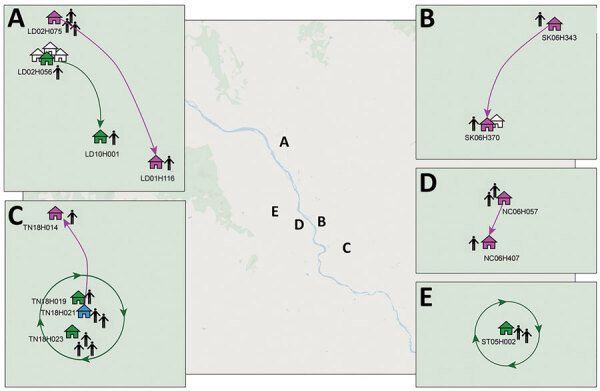
Approximate locations of dengue virus serotype 1 household transmission clusters and chains, Kamphaeng Phet, Thailand. Chains were confirmed by Bayesian consensus sequence (magenta houses) or minor variant (green houses) analyses or both methods (blue house). Households are described by their subdistrict, cluster, and house numbers, such that the first 2 letters denote subdistrict, the next 2-digit number denotes cluster, and an H followed by a 3-digit number denotes house number. LD, Lan Dokmai; NC, Nakhon Chum; SK, Sa Kaeo; ST, Song Tham; TN, Thep Nakhon.

We examined the distribution of sampling times within the 3 iSNV-confirmed transmission clusters to further characterize DENV transmission dynamics at this fine scale. All persons from transmission cluster 3 were sampled within the same day, suggesting that they were inoculated at about the same time (Table 2). The close household proximity and time of infection, together with identical virus consensus sequence and shared minor variants, would indicate infection from a common source and simultaneous minor variant spread to >6 different persons. In contrast, persons from transmission cluster 1 were sampled ≈2 weeks apart, a period that exceeds the known DENV incubation period and would have required transmission and preservation of minor variants through several bottlenecks across the invertebrate–vertebrate transmission cycle. By comparison, household transmissions confirmed by BEAST analyses only (no shared iSNVs) were on average 19 days apart (Table 1).

Finally, we used the transmission events confirmed by either Bayesian consensus sequence analysis (Table 1) or iSNV analyses (Table 2) to estimate the rate of DENV spread within subdistricts and between households. We performed a linear regression of transmission-pair distances over sampling time, with a model fit indicating a strikingly linear relationship (R^2^ = 0.91) and a regression coefficient indicating that DENV disperses an average of 70 m/day (95% CI 54–86 m/day) within subdistricts in this study population (Appendix Figure 4).

## Discussion

Dengue control approaches are best informed by granular characterization of the spatial epidemiology of these viruses. Even in active surveillance cohorts, however, resolution of transmissions on a household-scale is hampered by the limited geographic resolution permitted by case count, serologic, or even DENV whole-genome consensus sequence data. Our study leveraged a prospective active surveillance study with dense case sampling over 4 years, 410 whole-genome sequences and a unique combination of within-host and between-host viral genome variability to reconstruct transmission of DENV at unprecedented spatial scales. By using multiple lines of evidence, we tracked the transmission of DENV minor variants to trace DENV transmissions between households. Our findings emphasize the value of deep sequencing in resolving house-to-house transmission patterns, and offer a proof-of-concept approach to use intra-host DENV diversity for fine-scale case linkage as a public health tool. Our approach also resolved 3 transmission chains involving persons residing in the same households, providing direct genomic evidence for peridomestic transmission of DENV. We therefore present an evolutionary biology framework for the resolution of DENV transmissions that cannot be differentiated on the basis of epidemiologic and consensus genome data alone.

Our results also show that sharing of iSNV patterns is not guaranteed between epidemiologically linked cases sampled within a 2 week period. iSNVs might be lost because of bottlenecks, genetic drift, or individual immune responses. Therefore, the transmission dynamics of iSNVs are multifactorial, and iSNV-based case-linkage should be performed carefully in the context of complementary epidemiologic and consensus genome data. This point is emphasized by our detection of single iSNVs shared among multiple unrelated cases across entire DENV lineages, suggesting that identical single iSNVs might be stochastically found among unlinked cases. Distinguishing these stochastic iSNVs from transmission-related iSNVs is essential, highlighting the importance of inclusion of closely related background datasets, as done in our study. Only a small proportion of iSNVs in our sequenced cases could be attributed to transmission events, similar to the findings of Sim et al (*39*), and this finding emphasizes the complex evolutionary landscapes of arbovirus iSNVs. 

Recently, methods have been developed to take into account intra- and inter-host variation for analyses of pathogen spread; however, the limited number of iSNVs in DENV, short sequencing reads, and erroneous or shared iSNVs across unrelated taxa, might be limiting factors influencing the confidence of these tools (*40*–*42*). Our findings also offer a framework to determine functional DENV bottleneck sizes between sampled human dengue cases. Prior studies have shown that bottlenecks within the mosquito vectors themselves range from 5 to 42 genomes and that as many as 11.5% iSNVs are shared between suspected human–human transmission pairs (*39*,*43*). Such bottleneck estimates broadly fit with our observations of constellations of as many as 5 iSNVs shared between human cases sampled 14 days apart. However, long-read sequencing and larger sample sizes, as well as direct samples, from both vector and human populations would be required to definitively determine the number of distinct viral genomes that are passed through human–vector bottlenecks. Even though our genomes were derived from low-passage isolates, we did not observe a significant correlation between the number of passages or passage history and shared iSNVs (Appendix Table 1). Many iSNVs are preserved during low passage of DENV-1 clinical samples; nevertheless, in such instances, care must be taken along with use of appropriate statistics to ensure their accuracy (*44*).

The analyses of our confirmed consensus and minor variant transmission clusters further revealed a strong DENV spatial structure on an inter-household scale. Although household transmission pair distances varied from 0 to 3,000 m, our regression analysis of transmission pair distance over sampling time showed a strong linear relationship, indicating distance is a major determinant of DENV transmission at these ultra-fine spatial scales, and providing pilot estimates of the diffusion speed of DENV spread between households of 70 m/day (95% CI 54–86 m/day). Even though our regression analysis indicated a good model fit (R^2^ = 0.91), these coefficient estimates are nevertheless preliminary and are subject to potential nonindependence of transmission pair distance measurements and would benefit from confirmation in greater sample sizes in other study populations. This distance dependent dispersal between households was not noted in a prior Singapore study, which plotted uncorrected consensus genetic distance against geographic distance and sampling times (*21*). This finding highlights the importance of phylogenetic correction for genomic-based DENV tracing. Taken together, these findings support prior research that suggested that DENV transmissions are highly localized on this microspatial scale and might inform vector-control operations (*12*).

Although our findings were derived from an intensive, 4-year active cluster investigation across a very well sampled geographic area, ascertainment bias was a potential weakness in our study. All sequences were either derived from index case-patients accessing care for febrile illness or their contacts who were only sampled in households within 100–200 m of the index case-patient. However, our dense sampling of index cases over a 4-year study period ensured a large number of sequenced specimens across the Kamphaeng Phet region across multiple cluster investigations. Two of the 9 genetically confirmed transmission pairs were detected between persons who were not sampled in the same index case–contact cluster, highlighting the incremental value of genomic data over epidemiologic data alone in DENV transmission tracing. Future improvements in deep sequencing methods (*45*), coupled with more intensive vector and host sampling, could greatly improve our understanding of the invertebrate–vertebrate DENV iSNV bottleneck size and better resolve DENV vector–human transmission dynamics.

In conclusion, we have provided proof-of-concept evidence to support a new evolutionary epidemiologic framework that incorporates both within-host and between-host viral variation to determine fine-scale DENV transmissions. Our framework offers precision tracing of DENV at inter- and intra-household scales and leverages recent similar applications used for study of influenza and Ebola viruses (*23*,*24*). Our findings warrant larger studies in these and other dengue surveillance cohorts, as well as exploration of how this approach can be used for other arboviruses such as chikungunya and Zika viruses. Ideally, such studies should be coupled with advances in the accuracy of long-read deployable sequencing platforms that could permit near real-time intra-host variant construction (*46*).

Appendix Additional information about precision tracing of household dengue spread using inter- and intra-host viral variation data, Kamphaeng Phet, Thailand.
