# Supplementary material for: Precision Tracing of Household Dengue Spread Using Inter- and Intra-Host Viral Variation Data, Kamphaeng Phet, Thailand
Source: Emerg Infect Dis. 2021 Jun;27(6):1637–44. doi: 10.3201/eid2706.204323 (PMC8153871; doi:10.3201/eid2706.204323)
Supplement: Appendix — Additional information about precision tracing of household dengue spread using inter- and intra-host viral variation data, Kamphaeng Phet, Thailand. [file 20-4323-Techapp-s1.pdf]

Article DOI: https://doi.org/10.3201/eid2706.204323

Precision Tracing of Household Dengue Spread Using Inter- and Intra-Host Viral Variation Data, Kamphaeng Phet, Thailand

Appendix

Genomes sharing one iSNV only were positioned across the phylogeny, even between relatively unrelated viruses, with no significant difference between the frequencies of samples sharing a single iSNV within a transmission cluster versus across the sublineage ($p = 0.38$). However, there was a significant difference between the frequencies of samples sharing two or more iSNVs occurring within a transmission cluster versus across the sublineage ($p = 0.013$), indicating that the distribution of strains sharing $\geq$2 iSNVs within transmission clusters was not due to chance. In addition, the consensus sequence p-distance between the samples sharing two or more iSNVs was significantly lower compared to the p-distance between randomly sampled genomes from the DENV1 sublineages ($p<0.001$), indicating that the iSNV-sharing viruses were genetically significantly closer to each other than would be expected by chance. These results support the notion that these patterns of shared viral minor variants correlated with direct virus transmission chains and clusters. There was no significant correlation between the number of passages or passage history and shared minor variants (F-test, $p>0.05$) (Appendix Table 1).

Appendix Table 1. DENV1 sublineage 1 and 7 sample passaging history, with transmission clusters (TC) highlighted in blue, and samples with shared iSNVs in red text.

	D1 SL1	
Sample	Cell-passage no.	Transmission cluster
KDH0176A	C6/36–2	
KDH0168A	C6/36–2	
KDH0149A	C6/36–2	
KDH0131A	C6/36–3	
KDC0631A	C6/36–2	
KDH0142A	C6/36–3	
KDH0180A	C6/36–2	
KDH0163A	C6/36–2	
KDC0734A	C6/36–2	
KDH0154A	C6/36–2	
KDH0134A	TS-1,C6/36–1	
KDC0588A	C6/36–3	
KDC0587A	C6/36–3	
KDC0495A	C6/36–3	
KDH0151A	C6/36–2	
KDH0196A	TS-1,C6/36–1	
KDC0492A	C6/36–3	

D1 SL1		
Sample	Cell-passage no.	Transmission cluster
KDH0112A	TS-1,C6/36–1	
KDC0493A	C6/36–3	
KDH0143A	C6/36–2	
KDH0169A	TS-1,C6/36–1	
KDC0599A	C6/36–2	
KDC0501A	no info	
KDC0504A	no info	
KDH0132A	C6/36–3	
KDH0136A	TS-1,C6/36–1	
KDC0584A	C6/36–3	TC1
KDH0135A	C6/36–3	
KDH0129A	C6/36–3	
KDH0137A	TS-1,C6/36–1	
KDC0573A	C6/36–3	
KDC0574A	TS-1,C6/36–1	
KDC0574A2	TS-1,C6/36–1	
KDC0575A	C6/36–3	
KDH0110A	TS-1,C6/36–1	
KDC0455A	TS-1,C6/36–1	
KDC0454A	TS-1,C6/36–1	
KDH0102A	C6/36–3	
KDC0456A	C6/36–3	
KDH0100A	C6/36–3	

D1 SL7		
Sample	Cell-passage no.	Transmission cluster
KDH0081A	C6/36–3	
KDH0167A	C6/36–2	
KDC0722A	C6/36–2	
KDC1153A	C6/36–3	TC3
KDC1152A	C6/36–3	
KDC1151A	C6/36–3	
KDC1150A	C6/36–3	
KDC1149A	TS-1,C6/36–1	
KDC1148A	TS-1,C6/36–1	
KDH0309A	C6/36–3	
KDH0074A	TS-1, C6/36–1	
KDH0068A	C6/36–3	
KDC0394A	C6/36–3	
KDH0082A	C6/36–3	
KDH0087A	TS-1,C6/36–1	
KDH0073A	C6/36–4	
KDH0202A	TS-1,C6/36–1	
KDH0199A	C6/36–2	
KDH0198A	C6/36–2	
KDH0201A	TS-1,C6/36–1	TC2
KDC0885A	C6/36–2	
KDH0151A	C6/36–2	
KDH0160A	C6/36–2	
KDC0718A	C6/36–2	
KDC0640A	TS-1,C6/36–1	
KDH0144A	TS-1,C6/36–1	
KDH0194A	C6/36–3	

Appendix Table 2. BEAST parameters used for time-scaled inter-household spread analyses.

Serotype	Sublineage†	Nucleotide substitution model‡	Molecular clock model	Demographic model	Discrete trait (household) reconstruction model	MCMC length
DENV1	1	TrNef (TN93)	UCLN	Constant	CTMC, asymmetric	600 million
DENV1	7	TrNef (TN93)	UCLN	Constant	CTMC, asymmetric	1 billion
DENV2	2	GTR	UCLN	Constant	CTMC, asymmetric	600 million
DENV2	6	GTR+G+I	UCLN	Constant	CTMC, asymmetric	600 million
DENV2	11	TrNef (TN93)	UCLN	Constant	CTMC, asymmetric	600 million

†As classified in Appendix Figures 2 and 3.
‡Estimated by JModelTest2.

Appendix Table 3. Summary of probable and possible within-district household-to-household spread inferred by consensus genome discrete train Bayesian analysis†

Serotype	Sublineage	District	Origin household (probability)	Destination household (probability)	Level of evidence‡
D1	SL1	LD	LD02H002 (0.32)	LD02H033 (0.34)	Possible
D1	SL1	LD	LD02H033 (0.34)	LD02H001 (1.0)	Possible
D1	SL1	LD	LD02H002 (0.68)	LD02H008 (1.0)	Possible
D1	SL1	LD	LD02H002 (0.13)	LD01H001 (0.48)	Possible
D1	SL1	LD	LD01H001 (0.48)	LD02H075 (0.50)	Possible
D1	SL1	LD	LD02H075 (1.0)	LD01H116 (1.0)	Probable
D1	SL1	LD	LD02H075 (0.5)	LD02H056 (0.26)	Possible
D1	SL1	LD	LD02H056 (0.53)	LD02H085 (0.54)	Possible
D1	SL1	LD	LD02H085 (0.54)	LD10H001 (1.0)	Possible
D1	SL1	LD	LD02H084 (0.53)	LD02H021 (1.0)	Possible
D1	SL1	LD	LD02H056 (0.26)	LD02H084 (0.21)	Possible
D1	SL1	LD	LD02H084 (0.21)	LD10H044 (0.21)	Possible
D1	SL1	LD	LD02H084 (0.53)	LD10H021 (1.0)	Possible
D1	SL1	LD	LD02H044 (0.21)	LD10H042 (0.56)	Possible
D1	SL1	LD	LD10H042 (0.56)	LD02H098 (0.59)	Possible
D1	SL1	LD	LD10H044 (0.21)	LD10H043 (0.41)	Possible
D1	SL1	LD	LD10H043 (0.41)	LD10H044 (1.0)	Possible
D1	SL1	SK	SK10H028 (0.21)	SK12H001 (0.23)	Possible
D1	SL1	SK	SK12H001 (0.63)	SK11H001 (0.40)	Possible
D1	SL1	NM	NM24H098 (0.46)	NM24H107 (1.0)	Possible
D1	SL1	SK	SK12H001 (0.23)	SK10H081 (0.73)	Possible
D1	SL1	SK	SK10H081 (0.43)	SK06H346 (0.91)	Possible
D1	SL1	SK	SK06H346 (0.99)	SK06H370 (1.0)	Probable
D1	SL1	NC	NC09H028 (0.67)	NC09H029 (1.0)	Possible
D1	SL1	LD	LD01H073 (0.27)	LD01H051 (0.40)	Possible
D1	SL1	LD	LD01H051 (0.40)	LD01H037 (1.0)	Possible
D1	SL7	NC	NC12H016 (0.31)	NC12H001 (0.54)	Possible
D1	SL7	NC	NC12H016 (0.21)	NC06H057 (0.82)	Possible
D1	SL7	NC	NC12H001 (0.54)	NC04H179 (0.49)	Possible
D1	SL7	NC	NC06H057 (0.82)	NC06H407 (1.0)	Probable
D1	SL7	NC	NC04H179 (0.49)	NC10H001 (1.0)	Possible
D1	SL7	ST	ST05H002 (0.43)	ST04H001 (1.0)	Possible
D1	SL7	ST	ST05H002 (0.34)	ST12H072 (1.0)	Possible
D1	SL7	KT	KT03H049 (0.31)	KT03H371 (1.0)	Possible
D1	SL7	KT	KT03H054 (0.32)	KT03H049 (0.30)	Possible
D1	SL7	TN	TN18H021 (0.62)	TN18H023 (0.71)	Possible
D1	SL7	TN	TN18H023 (0.71)	TN18H019 (1.0)	Possible
D1	SL7	TN	TN18H021 (0.88)	TN18H014 (1.0)	Probable
D2	SL2	NB	NB06H055 (0.80)	NB06H084 (1.0)	Probable
D2	SL2	NP	NP08H080 (0.82)	NP08H044 (1.0)	Probable
D2	SL2	AT	AT12H032 (0.48)	AT12H026 (1.0)	Possible
D2	SL2	NP	NP07H043 (0.54)	NP07H051 (1.0)	Possible
D2	SL2	NB	NB09H001 (0.26)	NB09H026 (0.32)	Possible
D2	SL2	NB	NB09H026 (0.32)	NB09H004 (1.0)	Possible
D2	SL2	NB	NB09H026 (0.32)	NB09H008 (0.37)	Possible
D2	SL2	NB	NB09H008 (0.44)	NB09H026 (1.0)	Possible
D2	SL2	NB	NB09H008 (0.37)	NB21H030 (1.0)	Possible
D2	SL2	NP	NP07H319 (0.54)	NP08H001 (0.47)	Possible
D2	SL2	NP	NP08H001 (0.47)	NP08H080 (0.82)	Possible
D2	SL2	NB	NB10H001 (0.19)	NB06H046 (1.0)	Possible
D2	SL2	NB	NB10H001 (0.24)	NB18H015 (0.69)	Possible
D2	SL2	NB	NB18H015 (0.69)	NB18H091 (0.25)	Possible
D2	SL2	NB	NB18H091 (0.25)	NB10H040 (1.0)	Possible
D2	SL2	NB	NB18H001 (0.35)	NB06H073 (0.98)	Possible
D2	SL2	NB	NB18H001 (0.74)	NB01H028 (1.0)	Possible
D2	SL2	NB	NB10H001 (0.44)	NB10H079 (0.43)	Possible
D2	SL2	NB	NB18H079 (0.43)	NB18H054 (1.0)	Possible
D2	SL2	NB	NB18H079 (0.47)	NB10H001 (1.0)	Possible
D2	SL2	NB	NB18H001 (0.44)	NB18H089 (0.38)	Possible
D2	SL2	NB	NB18H089 (0.38)	NB06H056 (1.0)	Possible
D2	SL2	NB	NB18H089 (0.46)	NB06H055 (0.73)	Possible
D2	SL2	NB	NB06H055 (0.73)	NB10H068 (0.35)	Possible
D2	SL2	NB	NB06H068 (0.35)	NB10H099 (1.0)	Possible
D2	SL6	SK	SK06H790 (0.99)	SK06H485 (1.0)	Probable
D2	SL6	NB	NB18H030 (0.67)	NB04H086 (1.0)	Possible
D2	SL6	ST	ST06H010 (0.63)	ST02H030 (1.0)	Possible

Serotype	Sublineage	District	Origin household (probability)	Destination household (probability)	Level of evidence‡
D2	SL6	NM	NM25H016 (0.35)	NM03H095 (0.39)	Possible
D2	SL6	NM	NM03H095 (0.39)	NM19H055 (1.0)	Possible
D2	SL6	ST	ST06H010 (0.20)	ST04H032 (0.44)	Possible
D2	SL6	ST	ST04H032 (0.44)	ST04H043 (0.52)	Possible
D2	SL6	ST	ST04H043 (0.52)	ST07H013 (0.22)	Possible
D2	SL6	ST	ST07H013 (0.22)	ST07H003 (0.20)	Possible
D2	SL6	ST	ST07H003 (0.20)	STH7004 (0.72)	Possible
D2	SL6	ST	ST07H003 (0.31)	ST07H005 (1.0)	Possible
D2	SL6	ST	ST07H003 (0.57)	ST07H015 (1.0)	Possible
D2	SL6	ST	ST04H032 (0.57)	ST04H020 (0.79)	Possible
D2	SL6	ST	ST04H020 (0.79)	ST11H001 (0.48)	Possible
D2	SL6	ST	ST11H001 (0.48)	ST04H058 (1.0)	Possible
D2	SL6	ST	ST11H001 (0.77)	ST09H001 (0.62)	Possible
D2	SL6	ST	ST09H009 (0.36)	ST09H013 (1.0)	Possible
D2	SL11	TN	H05011055 (0.48)	H05111055 (1.0)	Possible
D2	SL11	NB	H01010063 (0.49)	H00610063 (1.0)	Possible
D2	SL11	NB	H00110063 (0.41)	H01010063 (0.49)	Possible

†Sublineages selected for analysis based on sufficient within-district sampling and adequate temporal structure (Appendix Figure 4)
‡Probable defined as origin household being directly ancestral to the destination household and both geographic states supported with a probability ≥0.8. Possible defined as origin household being directly ancestral to the destination household but with one or both geographic states having a probability <0.8.

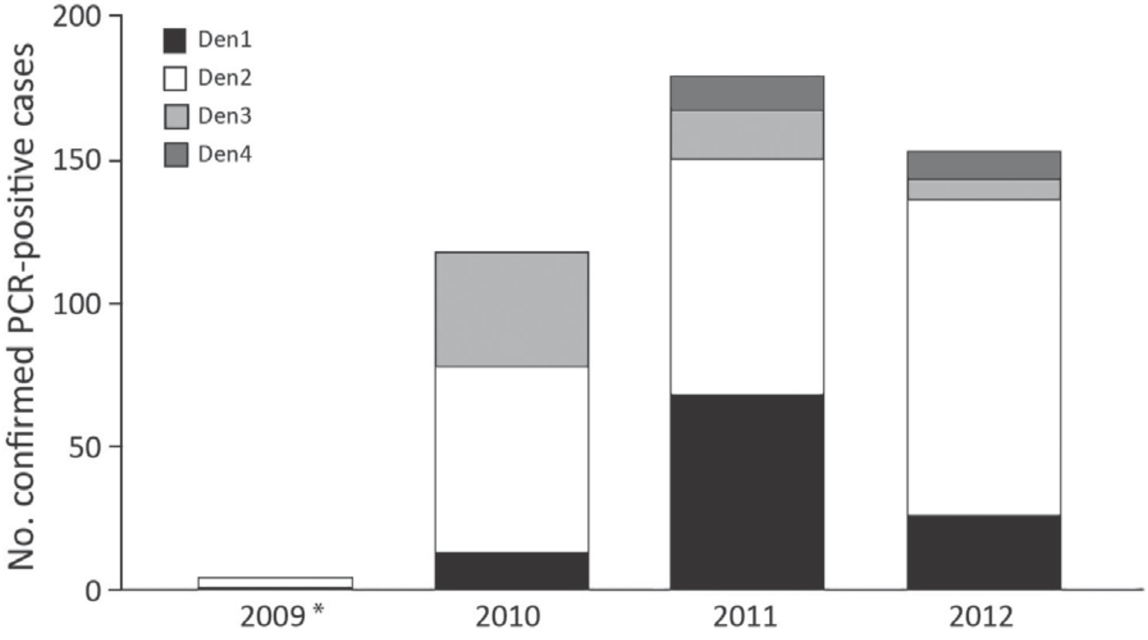

Appendix Figure 1. The number of PCR confirmed dengue cases by year and serotype collected between 2009 and 2012. 2009 samples were not collected across the full year.

A

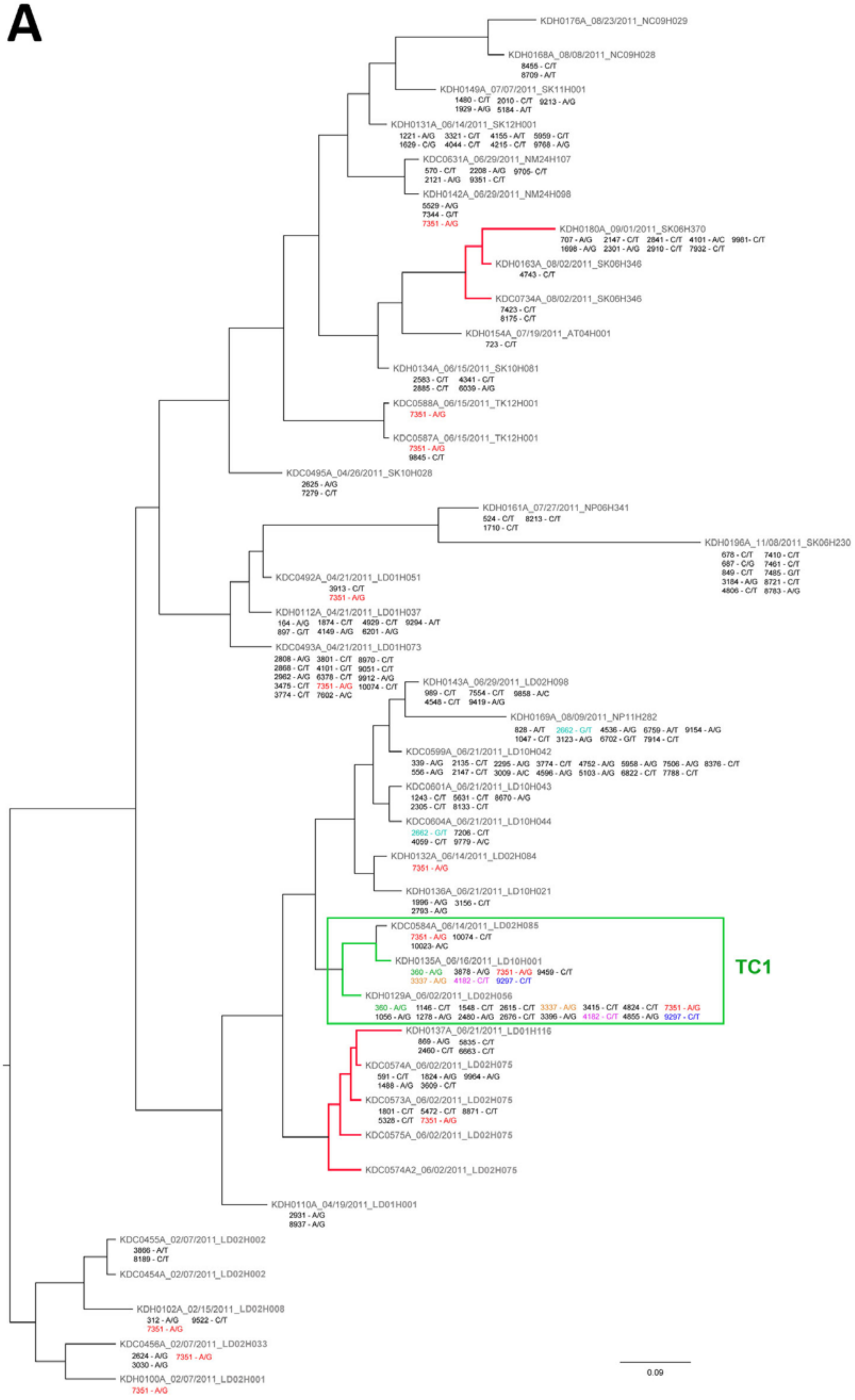

KDH0176A_08/23/2011_NC09H029
KDH0168A_08/08/2011_NC09H028
8455 - C/T
8709 - A/T
KDH0149A_07/07/2011_SK11H001
1480 - C/T 2010 - C/T 9213 - A/G
1929 - A/G 5184 - A/T
KDH0131A_06/14/2011_SK12H001
1221 - A/G 3321 - C/T 4155 - A/T 5959 - C/T
1629 - C/G 4044 - C/T 4215 - C/T 9768 - A/G
KDC0631A_06/29/2011_NM24H107
570 - C/T 2208 - A/G 9705- C/T
2121 - A/G 9351 - C/T
KDH0142A_06/29/2011_NM24H098
5529 - A/G
7344 - G/T
7351 - A/G
KDH0180A_09/01/2011_SK06H370
707 - A/G 2147 - C/T 2841 - C/T 4101 - A/C 9981- C/T
1698 - A/G 2301 - A/G 2910 - C/T 7932 - C/T
KDH0163A_08/02/2011_SK06H346
4743 - C/T
KDC0734A_08/02/2011_SK06H346
7423 - C/T
8175 - C/T
KDH0154A_07/19/2011_AT04H001
723 - C/T
KDH0134A_06/15/2011_SK10H081
2583 - C/T 4341 - C/T
2885 - C/T 6039 - A/G
KDC0588A_06/15/2011_TK12H001
7351 - A/G
KDC0587A_06/15/2011_TK12H001
7351 - A/G
9845 - C/T
KDC0495A_04/26/2011_SK10H028
2625 - A/G
7279 - C/T
KDH0161A_07/27/2011_NP06H341
524 - C/T 8213 - C/T
1710 - C/T
KDH0196A_11/08/2011_SK06H230
678 - C/T 7410 - C/T
687 - C/G 7461 - C/T
849 - C/T 7485 - G/T
3184 - A/G 8721 - C/T
4806 - C/T 8783 - A/G
KDC0492A_04/21/2011_LD01H051
3913 - C/T
7351 - A/G
KDH0112A_04/21/2011_LD01H037
164 - A/G 1874 - C/T 4929 - C/T 9294 - A/T
897 - G/T 4149 - A/G 6201 - A/G
KDC0493A_04/21/2011_LD01H073
2808 - A/G 3801 - C/T 8970 - C/T
2868 - C/T 4101 - C/T 9051 - C/T
2962 - A/G 6378 - C/T 9912 - A/G
3475 - C/T 7351 - A/G 10074 - C/T
3774 - C/T 7602 - A/C
KDH0143A_06/29/2011_LD02H098
989 - C/T 7554 - C/T 9858 - A/C
4548 - C/T 9419 - A/G
KDH0169A_08/09/2011_NP11H282
828 - A/T 2662 - G/T 4536 - A/G 6759 - A/T 9154 - A/G
1047 - C/T 3123 - A/G 6702 - G/T 7914 - C/T
KDC0599A_06/21/2011_LD10H042
339 - A/G 2135 - C/T 2295 - A/G 3774 - C/T 4752 - A/G 5958 - A/G 7506 - A/G 8376 - C/T
556 - A/G 2147 - C/T 3009 - A/C 4596 - A/G 5103 - A/G 6822 - C/T 7788 - C/T
KDC0601A_06/21/2011_LD10H043
1243 - C/T 5631 - C/T 8670 - A/G
2305 - C/T 8133 - C/T
KDC0604A_06/21/2011_LD10H044
2662 - G/T 7206 - C/T
4059 - C/T 9779 - A/C
KDH0132A_06/14/2011_LD02H084
7351 - A/G
KDH0136A_06/21/2011_LD10H021
1996 - A/G 3156 - C/T
2793 - A/G
KDC0584A_06/14/2011_LD02H085
7351 - A/G 10074 - C/T
10023- A/C
KDH0135A_06/16/2011_LD10H001
360 - A/G 3878 - A/G 7351 - A/G 9459 - C/T
3337 - A/G 4182 - C/T 9297 - C/T
KDH0129A_06/02/2011_LD02H056
360 - A/G 1146 - C/T 1548 - C/T 2615 - C/T 3337 - A/G 3415 - C/T 4824 - C/T 7351 - A/G
1056 - A/G 1278 - A/G 2480 - A/G 2676 - C/T 3396 - A/G 4182 - C/T 4855 - A/G 9297 - C/T
TC1
KDH0137A_06/21/2011_LD01H116
869 - A/G 5835 - C/T
2460 - C/T 6663 - C/T
KDC0574A_06/02/2011_LD02H075
591 - C/T 1824 - A/G 9964 - A/G
1488 - A/G 3609 - C/T
KDC0573A_06/02/2011_LD02H075
1801 - C/T 5472 - C/T 8871 - C/T
5328 - C/T 7351 - A/G
KDC0575A_06/02/2011_LD02H075
KDC0574A2_06/02/2011_LD02H075
KDH0110A_04/19/2011_LD01H001
2931 - A/G
8937 - A/G
KDC0455A_02/07/2011_LD02H002
3866 - A/T
8189 - C/T
KDC0454A_02/07/2011_LD02H002
KDH0102A_02/15/2011_LD02H008
312 - A/G 9522 - C/T
7351 - A/G
KDC0456A_02/07/2011_LD02H033
2624 - A/G 7351 - A/G
3030 - A/G
KDH0100A_02/07/2011_LD02H001
7351 - A/G
0.09

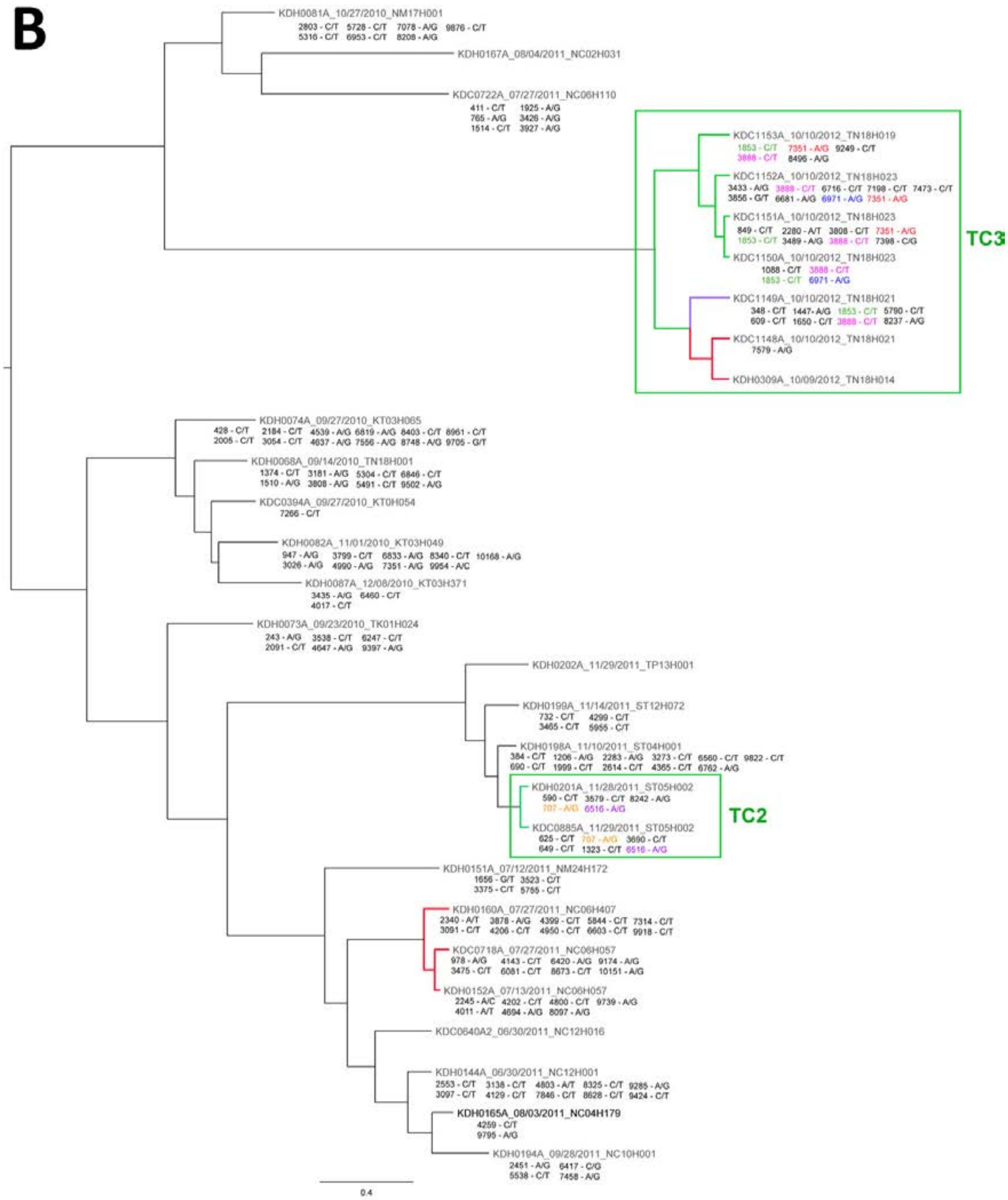

Appendix Figure 2. Maximum clade credibility trees of DENV1 A) Sublineage 1 and B) Sublineage 7, with households as discrete traits. BEAST confirmed transmission clusters/chains are marked red in the tree topology and minor variant confirmed ones are marked green. Purple branch indicates virus from an individual confirmed by both BEAST and minor variant analyses. The three transmission clusters detected by minor variant analyses are marked with green rectangles. All nucleotide positions containing minor variants and the variant composition of those positions are marked next to the respective taxon, shared minor variants are sharing the same color across taxons.

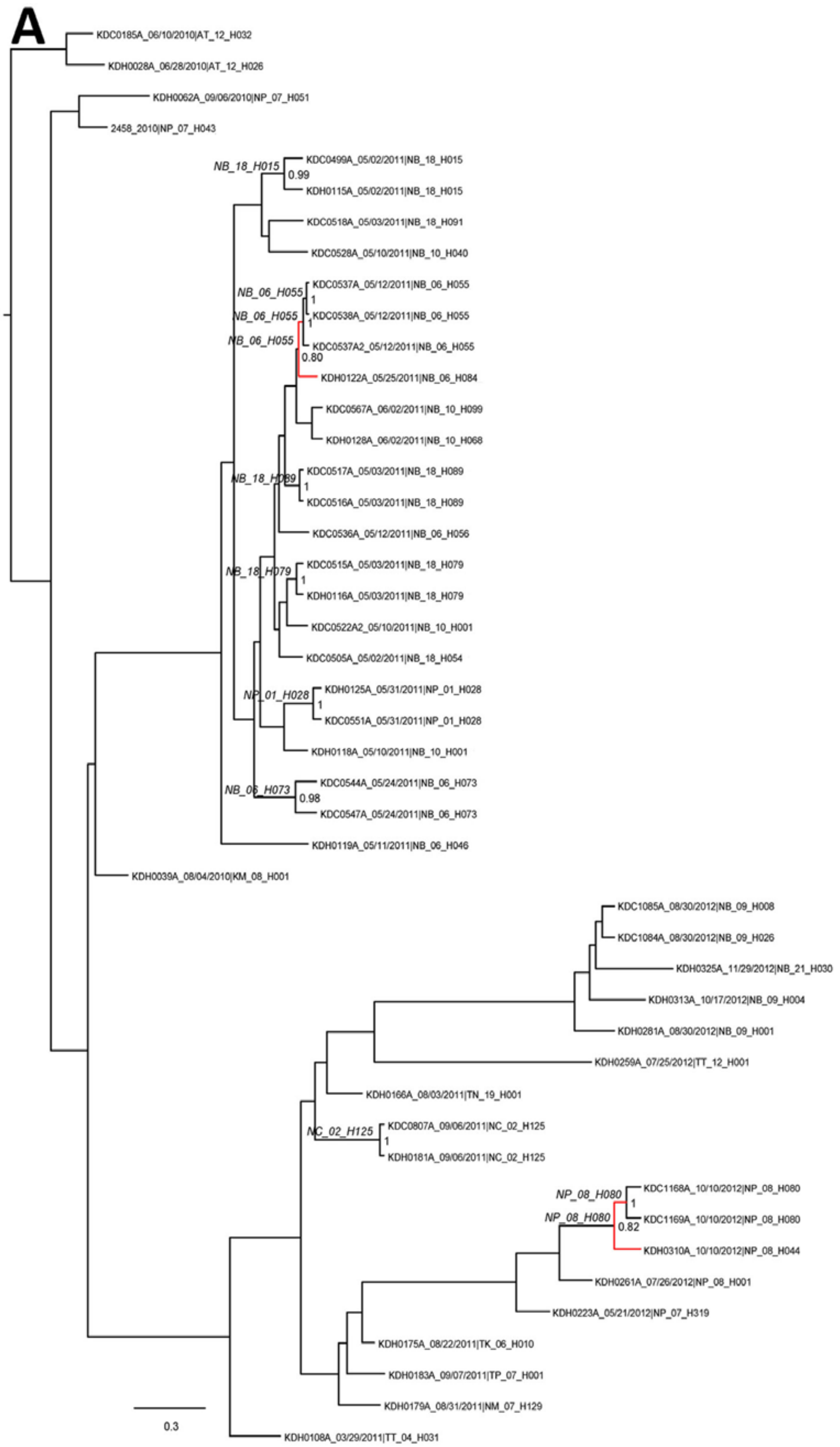
A
KDC0185A_06/10/2010|AT_12_H032
KDH0028A_06/28/2010|AT_12_H026
KDH0062A_09/06/2010|NP_07_H051
2458_2010|NP_07_H043
NB_18_H015
KDC0499A_05/02/2011|NB_18_H015
0.99
KDH0115A_05/02/2011|NB_18_H015
KDC0518A_05/03/2011|NB_18_H091
KDC0528A_05/10/2011|NB_10_H040
NB_06_H055
KDC0537A_05/12/2011|NB_06_H055
1
NB_06_H055
KDC0538A_05/12/2011|NB_06_H055
1
NB_06_H055
KDC0537A2_05/12/2011|NB_06_H055
0.80
KDH0122A_05/25/2011|NB_06_H084
KDC0567A_06/02/2011|NB_10_H099
KDH0128A_06/02/2011|NB_10_H068
NB_18_H089
KDC0517A_05/03/2011|NB_18_H089
1
KDC0516A_05/03/2011|NB_18_H089
KDC0536A_05/12/2011|NB_06_H056
NB_18_H079
KDC0515A_05/03/2011|NB_18_H079
1
KDH0116A_05/03/2011|NB_18_H079
KDC0522A2_05/10/2011|NB_10_H001
KDC0505A_05/02/2011|NB_18_H054
NP_01_H028
KDH0125A_05/31/2011|NP_01_H028
1
KDC0551A_05/31/2011|NP_01_H028
KDH0118A_05/10/2011|NB_10_H001
NB_06_H073
KDC0544A_05/24/2011|NB_06_H073
0.98
KDC0547A_05/24/2011|NB_06_H073
KDH0119A_05/11/2011|NB_06_H046
KDH0039A_08/04/2010|KM_08_H001
KDC1085A_08/30/2012|NB_09_H008
KDC1084A_08/30/2012|NB_09_H026
KDH0325A_11/29/2012|NB_21_H030
KDH0313A_10/17/2012|NB_09_H004
KDH0281A_08/30/2012|NB_09_H001
KDH0259A_07/25/2012|TT_12_H001
KDH0166A_08/03/2011|TN_19_H001
NC_02_H125
KDC0807A_09/06/2011|NC_02_H125
1
KDH0181A_09/06/2011|NC_02_H125
NP_08_H080
KDC1168A_10/10/2012|NP_08_H080
1
NP_08_H080
KDC1169A_10/10/2012|NP_08_H080
0.82
KDH0310A_10/10/2012|NP_08_H044
KDH0261A_07/26/2012|NP_08_H001
KDH0223A_05/21/2012|NP_07_H319
KDH0175A_08/22/2011|TK_06_H010
KDH0183A_09/07/2011|TP_07_H001
KDH0179A_08/31/2011|NM_07_H129
0.3
KDH0108A_03/29/2011|TT_04_H031

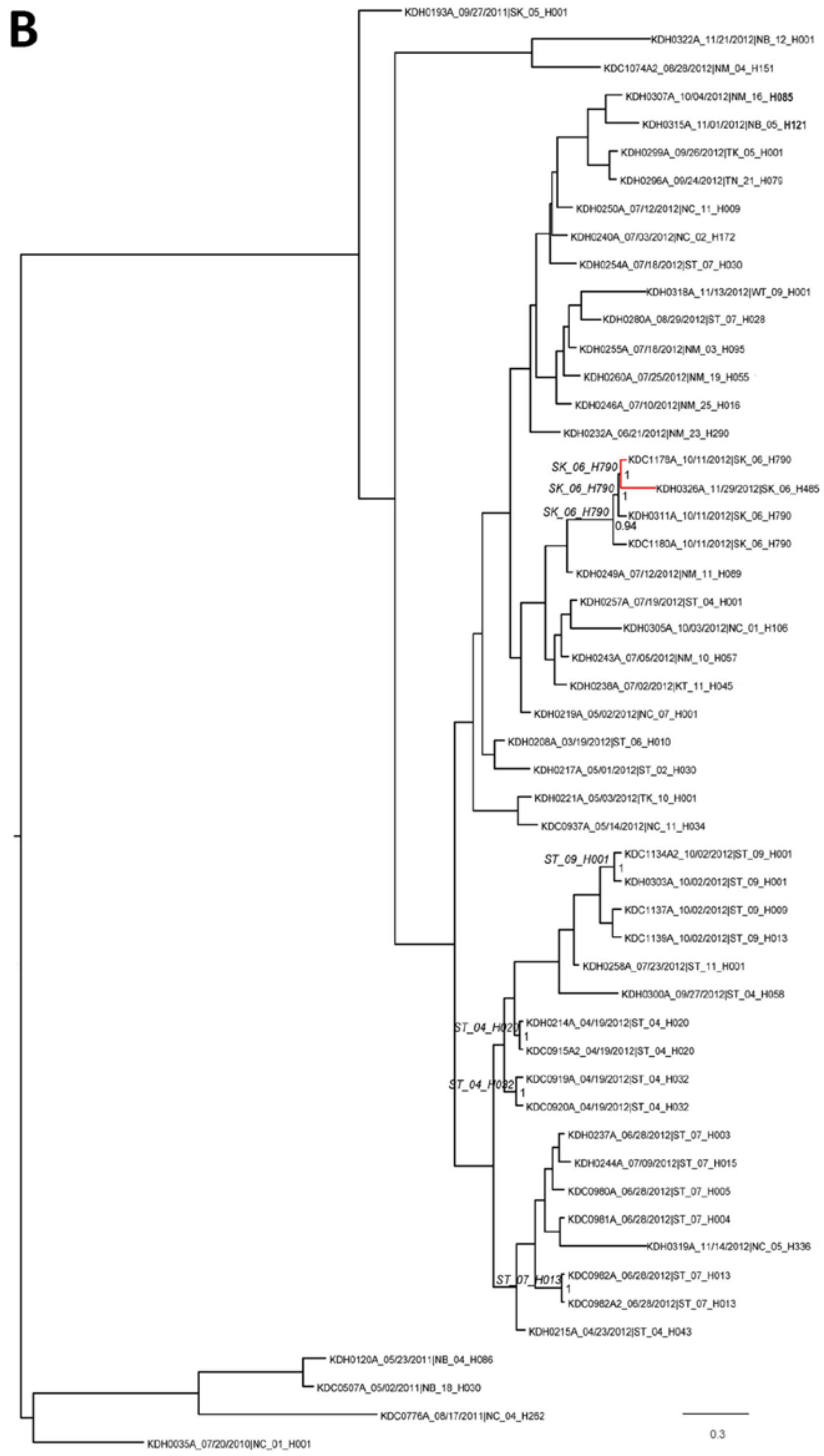

Appendix Figure 3. Maximum clade credibility trees of DENV2 A) Sublineage 2 and B) Sublineage 6, with households as discrete traits. BEAST confirmed transmission clusters/chains are marked red in the tree topology. 95% HPD geo location values >0.8 are shown next to the respective node, and the originating households are marked in italic font.

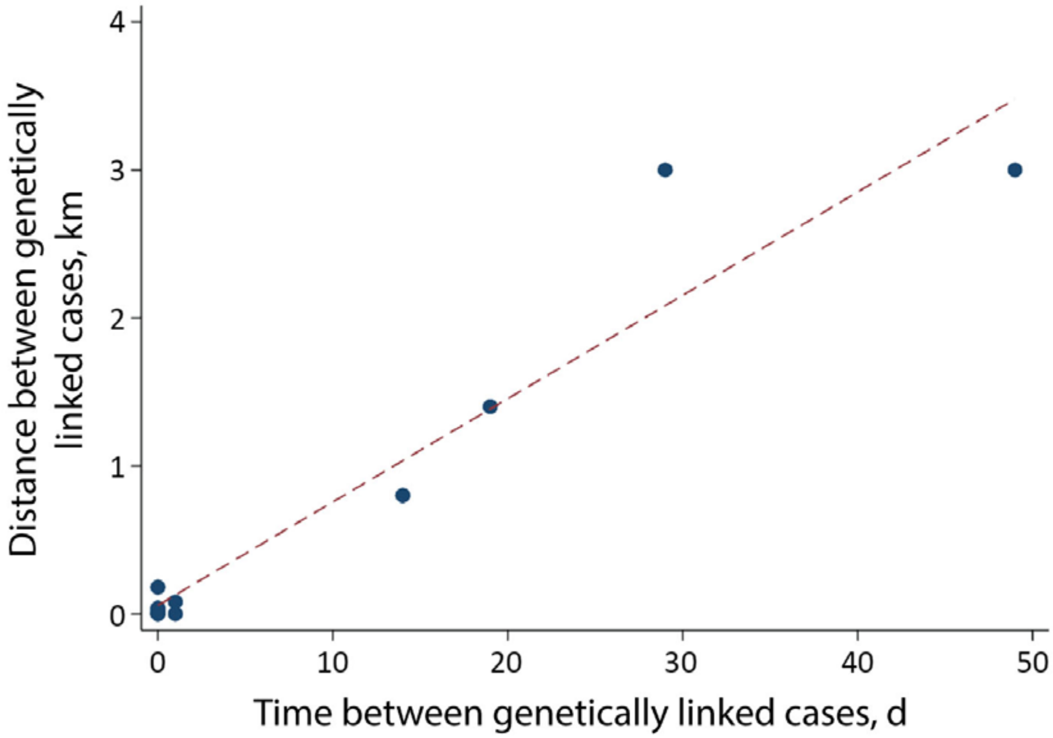

Appendix Figure 4. Linear regression of transmission-pair distances over sampling time.
